# Role of progression of training volume on intramuscular adaptations in patients with chronic obstructive pulmonary disease

**DOI:** 10.3389/fphys.2022.873465

**Published:** 2022-08-23

**Authors:** Andre Nyberg, Nadia Milad, Mickael Martin, Dany Patoine, Mathieu C Morissette, Didier Saey, François Maltais

**Affiliations:** ^1^ Institut Universitaire de Cardiologie et de Pneumologie de Québec, Université Laval, Québec, QC, Canada; ^2^ Department of Community Medicine and Rehabilitation, section of Physiotherapy, Umeå University, Umeå, Sweden

**Keywords:** COPD, resistance training, exercise, progression, muscle dysfunction, training volume

## Abstract

**Introduction:** Quadriceps dysfunction is a common systemic manifestation of chronic obstructive pulmonary disease (COPD), for which treatment using resistance training is highly recommended. Even though training volume is suggested to be a key explanatory factor for intramuscular adaptation to resistance training in healthy older adults, knowledge is scarce on the role of progression of training volume for intramuscular adaptations in COPD.

**Methods:** This study was a sub-analysis of a parallel-group randomized controlled trial. Thirteen patients with severe to very severe COPD (median 66 yrs, forced expiratory volume in 1 s 44% predicted) performed 8 weeks of low-load resistance training. In a *post hoc* analysis, they were divided into two groups according to their training volume progression. Those in whom training volume continued to increase after the first 4 weeks of training outlined the continued progression group (*n* = 9), while those with limited increase (<5%) or even reduction in training volume after the initial 4 weeks composed the discontinued progression group (*n* = 4). Fiber-type distribution and oxidative muscle protein levels, i.e., citrate synthase (CS), hydroxyacyl-coenzyme A dehydrogenase (HADH), mitochondrial transcription factor A (TfAM) as well as quadriceps endurance measures (total work from elastic band and isokinetic knee extension tests), were assessed before and after the intervention period.

**Results:** The continued progression group sustained their training volume progression during weeks 5–8 compared to weeks 1–4 (median +25%), while the discontinued progression group did not (median -2%) (*p* = 0.007 between groups). Compared with baseline values, significant between-group differences in fiber type distribution and TfAM muscle protein levels (range ± 17–62%, *p* < 0.05) and in individual responses to change in Type I and Type IIa fiber type proportion, CS, HADH, and TfAM muscle protein levels outcomes (median 89 vs. 50%, *p* = 0.001) were seen in favor of the continued progression group. Moreover, only the continued progression group had a significant increase in HADH muscle protein levels (+24%, *p* = 0.004), elastic band (+56%, *p* = 0.004) and isokinetic (+7%, *p* = 0.004) quadriceps endurance, but the between-group differences did not reach statistical significance (range 14–29%, *p* = 0.330–1.000).

**Discussion:** The novel findings of the current study were that patients with COPD who had a continued progression of training volume across the 8-weeks intervention had an increased proportion of Type I fibers, and TfAM muscle protein levels and decreased proportion of Type II fibers compared to those that did not continue to progress their training volume after the initial weeks. Additionally, HADH muscle protein levels and quadriceps endurance measurements only improved in the continued progression group, although no significant between-group differences were seen. These findings highlight the importance of continued progression of training volume during resistive training to counteract quadriceps dysfunction within the COPD population. Still, considering the small sample size and the *post hoc* nature of our analyses, these results should be interpreted cautiously, and further research is necessary.

## 1 Introduction

Quadriceps dysfunction is a common systemic manifestation of chronic obstructive pulmonary disease (COPD) (Maltais et al., 2014; Barreiro et al., 2015; Gea et al., 2015; Marklund et al., 2019). It results from numerous muscle structural, molecular, and cellular alterations, including weakness, atrophy, fiber-type distribution shifts, reduced oxidative capacity, and mitochondrial dysfunction, mediated by multiple etiological factors (Couillard and Prefaut, 2005; Maltais et al., 2014; Barreiro and Jaitovich, 2018; Jaitovich and Barreiro, 2018). The quadriceps is also more susceptible to fatigue when compared to healthy age-matched older adults, a situation that worsens with disease severity (Boccia et al., 2015; Boccia et al., 2016). Furthermore, quadriceps dysfunction is intimately linked with important clinical and prognostic outcomes, including exercise tolerance, daily physical activity, and even mortality (Swallow et al., 2007; Maltais et al., 2014; Frykholm, 2020; Géphine et al., 2021). Thus, counteracting quadriceps dysfunction is emphasized as a critical goal of COPD management (Spruit et al., 2013; Maltais et al., 2014; Marklund et al., 2019; Marillier et al., 2020), for which exercise training, specifically resistance training, is recommended over other available treatment modalities, irrespective of being performed alone or in combination with endurance training (Spruit et al., 2002; Troosters et al., 2010; Iepsen et al., 2015a; Iepsen et al., 2015b; Rinaldo et al., 2017; Jaitovich and Barreiro, 2018; Marklund et al., 2019).

Resistance training can be designed to enable various muscular adaptations (Kraemer and Ratamess, 2004; American College of Sports Medicine, 2009; Lopez et al., 2021). For example, it has been thought that an increased force or torque production could be expected following a muscle strength training intervention using higher loads and a lower number of repetitions, often described as high-load resistance training. In contrast, increased ability of the muscle to perform repetitive work may be more likely following resistance training using lower loads and a higher number of repetitions (often 15–25 RM or more), which is referred to as low-load resistance training (American College of Sports Medicine, 2009). Notably, recent research in healthy adults has highlighted that this repetition continuum is not as straightforward as initially thought, and both strength and endurance properties can be expected across the repetition continuum (Schoenfeld et al., 2017; Schoenfeld et al., 2021). Still, current evidence, although imperfect, indicates an advantage for low load resistance training to improve local muscle endurance of the lower extremity (Schoenfeld et al., 2021). Low-load resistance training has also recently been found to enable structural and morphologic muscle adaptations e.g., increased muscle citrate synthase activity (CS) and hydroxyacyl-coenzyme A dehydrogenase (HADH) muscle protein levels as well as increase capillary-to-fiber ratioconsistent with an increase in the endurance capacity of the quadriceps among patients with COPD, but without a clear effect on quadriceps strength (Nyberg et al., 2021). Similarly, among healthy adults, low-load resistance training has been found to increase quadriceps endurance and quadriceps oxidative capacity as evident by increased capillary contacts and capillary-to-fiber ratio (Pignanelli et al., 2019)

Nevertheless, irrespective of the type of resistance training modality used, training volume, commonly defined as total load lifted (sets × repetitions × external load) (Figueiredo et al., 2018; Brigatto et al., 2022), is considered to be a key explanatory factor for neuromuscular adaptation to exercise, with higher training volumes leading to larger effects (Naclerio et al., 2013; Figueiredo et al., 2018; Brigatto et al., 2022). Additionally, to enable continued muscle adaptations over time, progressive overload, that is the gradual increase of stress placed upon the muscles during exercises (Kraemer and Ratamess, 2004; American College of Sports Medicine, 2009), is of utmost importance. Notably, progressive overload can be accomplished by a gradual increase of training volume (Kraemer and Ratamess, 2004; American College of Sports Medicine, 2009), which will drive neural and intramuscular adaptations and in turn allow the patient to tolerate a higher training volume and hence increase the effect of resistance training over time (Sale, 1988; Peterson et al., 2004; Peterson et al., 2011; Škarabot et al., 2021).

The effects of resistance training on muscle function over time can be explained by a combination of neural (Gabriel et al., 2006; Jenkins et al., 2017; Škarabot et al., 2021)) and intramuscular adaptations (Sale, 1988; Folland and Williams, 2007; Škarabot et al., 2021)). During a typical 8-weeks training period, it has been suggested that neural adaptations account for up to 90% of gain in muscle function during the first 2–3 weeks of training, 40–50% during the following weeks, while the relative importance of muscular adaptations, such as morphological and structural alterations on muscle function improvements increases over time (Sale, 1988; Folland and Williams, 2007; Škarabot et al., 2021). These latter findings suggest that continued progression of training volume after the initial weeks of training is likely needed for intramuscular adaptations to occur. If not, an improved muscle function would likely result mainly from neural and not intramuscular adaptations (Sale, 1988; Folland and Williams, 2007; Škarabot et al., 2021). Thus, optimal training volumes and specifically a continued progression of training volume over time are likely needed if the goal is to counteract limb muscle dysfunction in COPD, enabling structural and morphological muscle adaptations (Maltais et al., 2014; Barreiro et al., 2015; Barreiro and Gea, 2015). However, knowledge is scarce on the importance of training volume and, specifically progression of training volume for intramuscular adaptations to occur after resistive training in patients with COPD. The primary aim of this study was to determine the role of progression of training volume on limb muscle adaptations in a sample of patients with COPD who were involved in a clinical 8 week trial of low resistance/high repetition resistance training, taking advantage of the fact that the progression of training was variable amongst the study participants. The focus of our comparison will be on intramuscular, not neural adaptations, thus we specifically target progression of training volume after the initial weeks of training. Based on previous findings and recommendations (Sale, 1988; Peterson et al., 2004; American College of Sports Medicine, 2009; Peterson et al., 2011; Škarabot et al., 2021), we hypothesized that intramuscular adaptations would be more profound in those with a continued progression of training volume than in those in whom training volume reached a plateau after the first few weeks of training.

## 2 Materials and methods

### 2.1 Study design

The present report is a *post-hoc* analysis of a prospective, assessor- and statistician blind; randomized controlled, parallel-group trial (Nyberg et al., 2015; Nyberg et al., 2021) constructed following the Consolidated Standards of Reporting Trials guidelines (Moher et al., 2010) whose objective was to evaluate the effects of low-load/high resistance training on exercise capacity, health status, and limb muscle adaptation in COPD . The study involved patients with COPD who performed 8-weeks of low-load resistance training designed to increase the endurance capacity of the quadriceps (Nyberg et al., 2021). Per study design, the training volume progressed according to individual’s tolerance. Because of this, the progression of training varied from one individual to the other, allowing to study the impact of the progression of training volume on limb muscle adaptations. Participants were initially randomized to single-limb or two-limb execution of resistance exercises (Nyberg et al., 2021). However, for the purpose of this secondary analysis participants were retrospectively divided into two groups according to their training volume progression independent of initial randomization. Those in whom training volume continued to increase after the first 4 weeks of training (>5%) outlined the continued progression group (*n* = 9), while those with limited increase (≤5%) or even decrease in training volume after the initial 4 weeks composed the discontinued progression group (*n* = 4). The 5% threshold was based on the American College of Sport Medicine guidelines for progressive overload in resistance training for healthy adults (Kraemer and Ratamess, 2004; American College of Sports Medicine, 2009). The local ethics committee approved the protocol (CER:21,111), and the trial was prospectively registered at www.clinicaltrials.gov (NCT02283580). Detailed descriptions of study procedures have been published elsewhere (Nyberg et al., 2015).

### 2.2 Participants

Inclusion criteria were stable COPD, ≥ 40 years of age with a post-bronchodilator forced expiratory volume in 1 s (FEV_1_) < 50% of predicted value (i.e., GOLD stage 3–4), and a cumulative (current or ex) smoking history >10 pack-years. The exclusion criteria for participation in the trial were; recent COPD exacerbation (<6 weeks), neuromuscular or orthopedic disorders that compromise involvement in an exercise program, recent cancer, unstable cardiac disease, and cardiac stimulator, asthma (current), low body weight or obesity (body mass index < 20 kg/m^2^ or > 30 kg/m^2^), significant hypoxemia at rest (SpO_2_ < 85%), or a daily dose > 10 mg of oral prednisone. In addition, specific for this sub-analysis, we have only included participants who had consented to a muscle biopsy before and after an 8-weeks intervention consisting of low-load resistance training designed per American College of Sport Medicine recommendations for improving the endurance capacity of the target muscle (American College of Sports Medicine, 2009).

### 2.3 Outcome measures

Outcome assessment was performed before and after the 8-weeks training program. Weight, height, fat-free mass (Kg and percentage), and right leg weight were measured. In addition, pulmonary function, self-reported physical activity (Voorips questionnaire)(Voorrips et al., 1991), exercise capacity (6MWD (Wise and Brown, 2005)), and health status (COPD assessment test [CAT] (Jenkins et al., 2017)) were assessed and used to describe the patient population.

#### 2.3.1 Quadriceps function

Isokinetic endurance of the quadriceps was measured using a protocol consisting of 30 maximal contractions at an angular velocity of 90°/second using an isokinetic dynamometer (Biodex Multi-Joint System 4, Biodex Corp., Shirley, New York). The range of movement in the knee joint was set from 90° to maximal individual extension minus 5° to lower the risk for a passive resistance from the hamstrings muscles. During the test, strong verbal encouragement was given for every contraction to contract in the concentric phase of the movement when extending the leg and to relax in the eccentric phase when getting back to the start position. Outcome measure was total work of all 30 contractions reported in Joules. In addition, elastic knee extension endurance was evaluated by comparing the work performed during the first set between the first and last exercise session (session # 24). Elastic work was measured during the first set from the number of repetitions multiplied by the load provided by the elastic band ([Kg] at 100% elongation)(Nyberg et al., 2021). Importantly, isokinetic and elastic assessments were performed using valid and reliable test procedures for COPD (Evans et al., 2014; Nyberg et al., 2018; Frykholm et al., 2019; Frykholm, 2020; Géphine et al., 2021).

#### 2.3.2 Muscle biopsies

Before and after the 8-weeks program a needle biopsy of the vastus lateralis was performed as described by Bergström and routinely done in our laboratory (Maltais et al., 2000; Doucet et al., 2010). At baseline, the biopsy was performed on a separate visit and before the training program commenced, while a minimum of 48 h of rest was allowed between the last exercise session and the biopsy performed at the end of program. The muscle biopsy specimens were immediately embedded in optimal cutting temperature (OCT) compound (Tissue Tek, Miles, Elkhar, IN, United States) and rapidly frozen in liquid nitrogen-cooled with isopentane. Vastus lateralis muscle analyses included typology, enzymatic and mitochondrial activities, and capillarization. We investigated the fiber-type proportion for each fiber type (Type I, Type IIa, Inter I/IIa, and IIb as well as protein levels of mitochondrial transcription factor A (TfAM) and hydroxyacyl coenzyme A dehydrogenase (HADH) and citrate synthase (CS) protein levels.

##### 2.3.2.1 Immunohistochemistry and antibodies

Muscle samples were cut in a cryostat in 10 μm serial cross-sections, mounted on glass slides, and stained with previously characterized antibodies to demonstrate muscle fiber types and visualize the muscle cells' basement membrane. In brief, the sections were then incubated for 1 hour at room temperature with primary mouse IgM anti-MyHC I (A4.840, Developmental Studies Hybridoma Bank (DSHB) at the University of Iowa, Iowa City, IA, United States), includeed mouse IgG anti-MyHC IIA (SC-71, DSHB, and rabbit IgG anti-laminin (Z0097, Dako). Muscle sections were then washed in phosphate-buffered saline (PBS) and mounted using PermaFluor (Fisher Scientific)**.** For visualization of capillaries, muscle cross-sections were fixed for 15 min in cold methanol at -20°C and rehydrated in PBS for 10 min at room temperature. A steamer achieved antigen retrieval using a citrate buffer (pH 6.0) for 20 min. Following quenching and protein blocking steps provided by the secondary antibody kit, slides were incubated with primary anti-CD31 antibody (ab9498) or IgG1 isotype control antibody (ab91353) from Abcam (Cambridge, United Kingdom) diluted 1:200 in PBS with 1% BSA overnight at 4°C. After three washes with PBS, secondary antibody incubation and HRP revelation were performed using the HRP/DAB Detection kit (ab64264; Abcam, Cambridge, United Kingdom) following the manufacturer’s instructions.

##### 2.3.2.2 Muscle fiber type classification, immunoblotting and morphometric analysis

Based on the immunostaining pattern for the different MyHC fiber types, muscle fibers were classified as slow MyHC-I (type I), fast MyHCIIa (type IIa), intermediate slow/fast MyHC I + IIa (type I/IIa), or fast MyHC IIx (type IIb). Muscle biopsies were homogenized in RIPA buffer with protease and phosphatase inhibitors, sonicated on ice three times for 5 s, and protein was extracted by centrifugation at 10,000g for 10 min at 4°C. Protein quantitation was performed using the DC Protein Assay (#5000116; Bio-Rad, Mississauga, ON, Canada), and 25 μg of protein for each sample was loaded on separate gels for each protein of interest: mitochondrial transcription factor A (TfAM; 25 kDa, 12% gels), citrate synthase (45 kDa, 12% gels) and hydroxyacyl coenzyme A dehydrogenase (HADH; 74 kDa, 10% gels). Muscle samples and internal control were run for 25 min at 50V, then 1.5 h at 110V, and transferred to a nitrocellulose membrane for 1–1.5 h at 100V 4°C. Membranes were incubated with Revert Total Protein Stain (#926–11010; LI-COR Biosciences, Lincoln, NE, United States) for 5 min to quantify total protein per lane. Membranes were blocked for 1 hour at room temperature with 5% skim milk in TBS-T. Next, membranes were incubated overnight at 4°C with rabbit monoclonal antibodies diluted in 5% BSA in TBS-T at the following concentrations: TfAM (1:1,000, D5C8; Cell Signaling, Danvers, MA, United States), citrate synthase (1:1,000, D7V8B; Cell Signaling, Danvers, MA, United States) and HADH (1:1,000, ab203114; Abcam, Cambridge, United Kingdom). Membranes were washed three times in TBS-T and then incubated with IRDye800 goat anti-rabbit secondary antibody (1:10,000, #926–32211; LI-COR Biosciences, Lincoln, NE, United States) diluted in 5% skim milk in TBS-T. After two washes in TBS-T and a final wash in TBS, images were taken using the Odyssey system (LI-COR Biosciences, Lincoln, NE, United States). Band intensities were normalized to the internal control sample on each gel and each sample’s total protein quantity. For the morphometric analysis of the muscle, images were captured on a fluorescent microscope. Fiber-type proportions (Type I, Type IIa, type I/IIa, and type IIb) were determined for each fiber type with the ImageJ software (United States National Institutes of Health, Bethesda, Maryland, United States).

### 2.4 Exercise interventions

All participants performed low-load resistance training, extensively described elsewhere (Nyberg et al., 2015; Nyberg et al., 2021) In brief, the low-load- resistance training regimen consisted of a total of seven individual exercises, each performed during two sets, three times per week for 8 weeks, with each set performed until voluntary failure. Exercises order was predetermined to alternate between agonist-antagonist in the following order: knee extension, (m. quadriceps), leg curl, (m. hamstrings), latissimus row, (m. latissimus dorsi), chest press, (m. pectoralis major, m. deltoid anterior), elbow flexion, (m. biceps brachii), shoulder flexion, (m. deltoid anterior), and calf-raises (mm. triceps surae).

**FIGURE 1:**
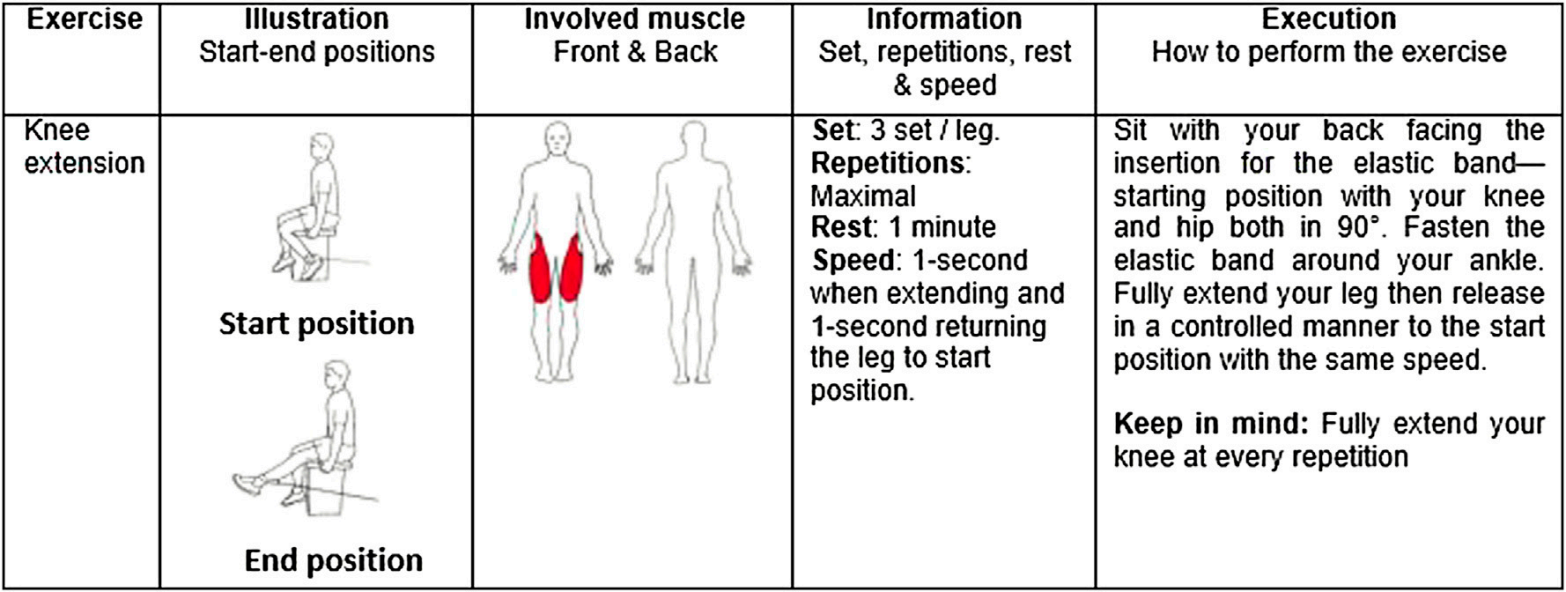
Description of the knee extension exercise.

The initial load for the knee extension exercise was individually set. It corresponded to the maximum load that could be lifted a minimum of 20 repetitions but a maximum of 30 repetitions). The 20–30 repetition maximum zone was based on resistance training guidelines for improving local muscle endurance (Kraemer and Ratamess, 2004; American College of Sports Medicine, 2009; Lopez et al., 2021). Progression of training volume was made based on the following criteria: when a participant performed > 30 repetitions in the first set of two subsequent sessions or two out of three successive sessions of an exercise, the intensity (loading) of that exercise was increased with approximately by 10% by changing the tension of the elastic band(s). Using the same criteria, the intensity was decreased if < 20 repetitions were performed (Nyberg et al., 2015; Nyberg et al., 2021).The knee extension exercise was performed with elastic bands (Thera-Bands, The Hygenic Corporation 1,245 Home Ave. Akron, OH 44310). For the purpose of the knee extension exercise, participants were positioned so that elastic bands would be stretched 100% of its initial length at the end of motion (Figure 1). Six different elastic bands (Yellow: 1.3 Kg [at 100% elongation], Red: 1.8 Kg, Green: 2.3 Kg, Blue: 3.2 Kg, Black: 4.4 Kg, Silver: 6.0 Kg) were used in isolation or in combination to achieve adequate resistances. For the purpose of this study, only the knee extension exercise was considered since it was the only exercise that targeted the quadriceps. The number of attended sessions was used to examine adherence.

### 2.5 Statistical analysis

Statistical analyses were performed with Jamovi (Version 1.6) [Computer Software]. Nonparametric Wilcoxon rank-sum and Mann-Whitney U tests were used for all analyses per guidelines for small sample sizes (Dwivedi et al., 2017). Data was compared between those in whom training volume continued to increase after the first 4 weeks of training (continued progression group) and those with reduced, no, or limited increase in training volume after the initial 4 weeks (discontinued progression group). In addition, the difference in the proportion of individuals showing an increase in the percentage of Type I fibers, an increase in TfAM, CS, HADH muscle protein levels, or a reduction in the proportion of Type IIa fibers that could be expected following the low-load design (American College of Sports Medicine, 2009) was examined through Fisher’s exact test. In the latter analysis, data was dichotomized before analysis according to the presence or not of intramuscular adaptation according to the following criteria: a change needed to be seen in at least four out of five muscle variables after the training intervention to be considered a responder. Spearman Rho (r_s_) was thereafter used to investigate possible associations between differences in training volume between the first and the last 4 week period and changes in muscle variables. Associations (r_s_) in the range of < 0.1 was considered trivial, 0.1–0.3 small, 0.3–0.5 moderate, 0.5–0.7 large, 0.7–0.9 very large, and > 0.9 extremely large (Hopkins et al., 2009). Data are presented as median (interquartile range [IQR]) or percentages (%), if otherwise not stated. Alpha was set at 0.05 for all analyses.

## 3 Results

### 3.1 Progression of training volume

**FIGURE 2:**
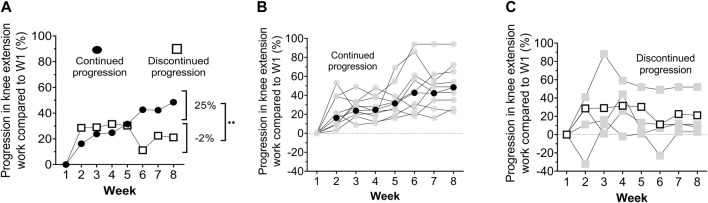
Mean percentage increase in knee extension training volume compared to week one among those with continued progression of training volume (continued) and those with reduced, no, or limited increase in training volume after the initial 4 weeks (discontinued), respectively **(A)**.% increase in training workloads during week 5–8 compared to week 1–4. Individual (light grey) and mean (black circles/white boxes) values in the continued group **(B)**, and the discontinued group **(C)**, respectively W = week. * *p* < 0.05, ** *p* < 0.001.

In total, a muscle biopsy was obtained in thirteen individuals with COPD before and after the 8-weeks low-load resistance training intervention program and were included in the present sub-analysis. At baseline, no statistically significant differences in anthropometrics, lung function, exercise capacity, fiber-type distribution, or oxidative muscle protein levels were seen between groups, even though participants in the continued progression group tended to be older than those in the discontinued progression group (69 vs. 60 years, *p* = 0.053) (Table 1). There was no difference in the median amount of attend sessions between groups (98% vs 96%, *p* = 0.429). As seen in Figure 2A, the continued progression group had increased training volume during week 5–8 compared to week 1–4 (median difference +25%), while the discontinued progression group did not continue to increase training volume after the initial 4-week period (median difference -2%), (*p* = 0.007 between groups). Total training volume was not different between the two groups (+3%, *p* = 0.825), despite the difference in progression between the continued- and discontinued progression groups.

**TABLE 1:** Patient characteristics.

	Continued progression (*n* = 9)	Discontinued progression (*n* = 4)	*p*
Anthropometrics			
Age, yrs	69 (66–70)	60 (56–64)	0.053
Weight, Kg	71 (66–78)	78 (70–81)	0.503
Height, cm	168 (165–169)	168 (161–174)	0.938
BMI, kg/m^2^	27 (25–27)	25 (25–27)	0.817
FFM, Kg	46 (42–51)	51 (43–55)	0.710
FFM, %	66 (64–70)	64 (60–70)	0.710
Right leg weight (Kg)	7.1 (6.2–7.6)	7.5 (6.5–8.4)	0.825
Lung function			
FVC, L	2.9 (2.5–3.3)	3.3 (2.1–4.3)	0.940
FVC, % predicted	77 (71–89)	89 (80–101)	0.330
FEV_1_, L	1.0 (0.8–1.2)	1.3 (0.9–1.6)	0.503
FEV_1_, % predicted	42 (28–46)	45 (39–48)	0.440
FEV_1_/FVC, %	36 (31–40)	37 (35–40)	0.710
MVV, L	38 (31–48)	53 (35–65)	0.503
Exercise capacity/muscle function			
6MWT, m	449 (404–470)	434 (400–472)	0.825
Isokinetic knee endurance, joule	2813 (2374–3,496)	3,976 (2991–4,821)	0.414
Elastic knee endurance, kg	240 (176–365)	263 (229–305)	0.940
Fiber type distribution			
Type I, %	29 (22–33)	31 (24–36)	0.940
Type IIa, %	67 (65–72)	57 (50–69)	0.260
Type I/IIa, %	1.5 (1.2–1.6)	5.2 (2.7–11)	0.685
Type IIb, %	0.5 (0.0–1.8)	0.2 (0.0–1.0)	0.214
Oxidative muscle protein levels			
CS	1.76 (1.50–2.03)	1.72 (1.58–1.77)	0.604
TfAM	2.11 (1.07–2.25)	1.62 (1.22–2.73)	0.940
HADH	0.96 (0.89–1.03)	0.89 (0.73–1.06)	1.000

Data is median (interquartile range 25–75). *p* < 0.05. BMI, body mass index, FFM, fat free mass, FVC, forced vital capacity, FEV_1_, forced expiratory volume at 1 s, MVV, maximum voluntary ventilation, 6MWT, 6-min walk test, CS, citrate synthase; HADH, hydroxyacyl-coenzyme A dehydrogenase; TfAM, mitochondrial transcription factor A.

### 3.2 Intramuscular adaptations

#### 3.2.1 Fiber type distribution and muscle protein levels

**FIGURE 3:**
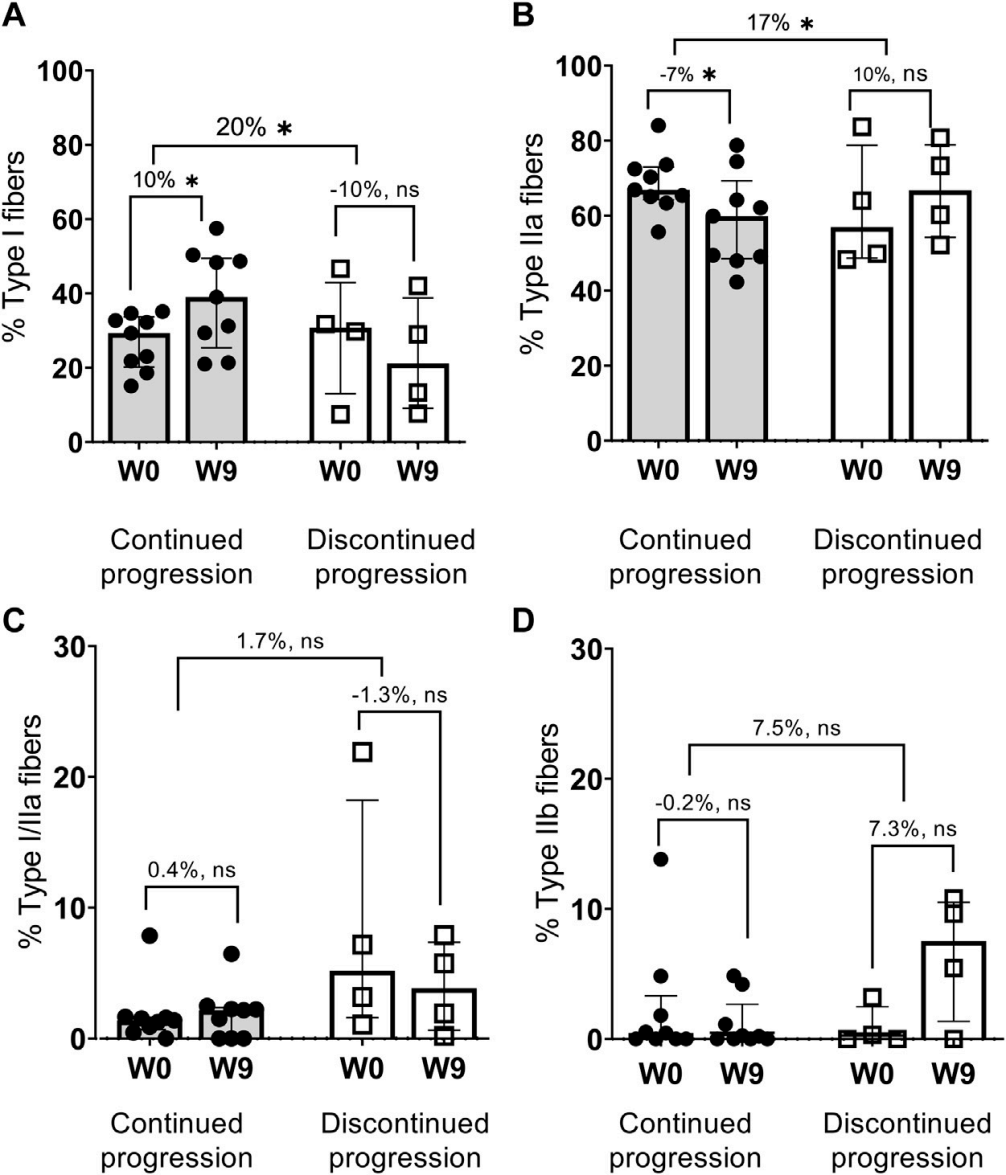
Changes in fiber type distribution, % Type I fibers **(A)**, % Type IIa fibers **(B)**, % Type I/IIa fibers **(C)**, % Type IIb fibers **(D)** * *p* < 0.05 (Bold).

**FIGURE 4:**
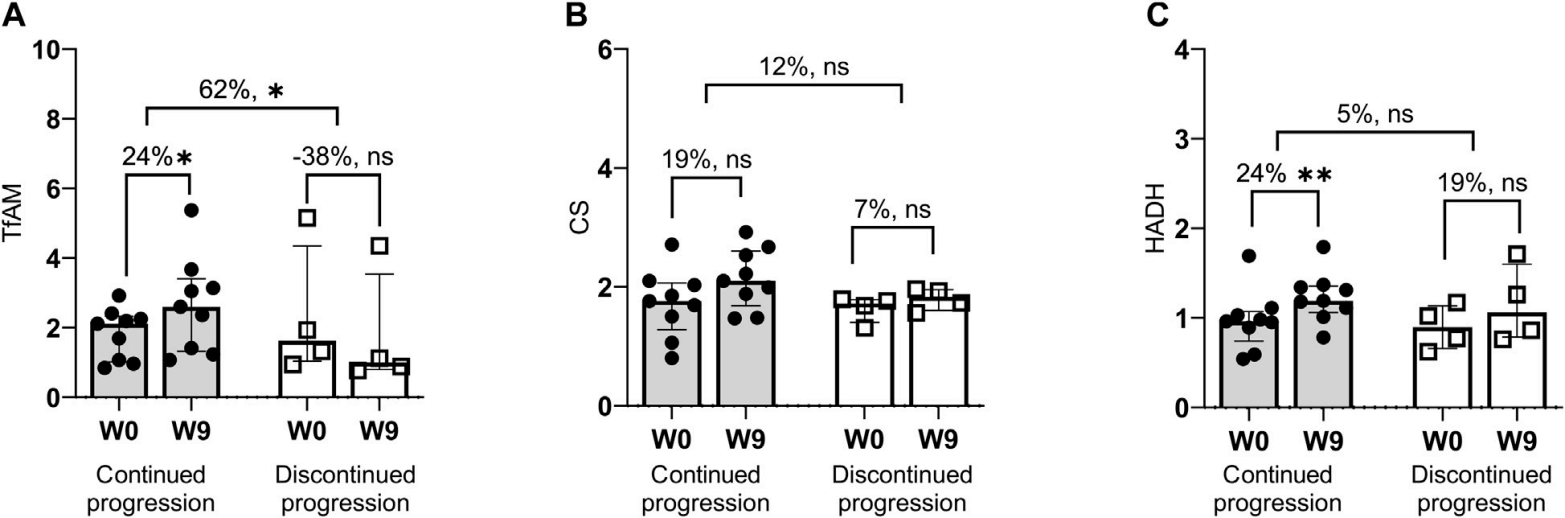
Changes in mitochondrial (oxidative) enzymes. Median values with error bars (75% interquartile range). TfAM = mitochondrial transcription factor A **(A)**; CS, citrate synthase **(B)**; HADH = hydroxyacyl-coenzyme A dehydrogenase **(C)**, W, Week. * *p* < 0.05, ** *p* < 0.001.

Compared with baseline values, the median between-group difference in the percentage of Type I (20%) and Type IIa (17%) fibers (Figure 3), as well as TfAM protein levels (62%) (Figure 4) was in favor of the continued progression group. Additionally, significant changes in TfAM and HADH muscle protein levels were seen in the continued progression group (+24, *p* < 0.05) but not in the discontinued progression group (*p* > 0.05). However, between-group differences for these variables did not reach statistical significance (Figure 4).

#### 3.2.2 Fiber type distribution and muscle protein levels–individual responses

**FIGURE 5:**
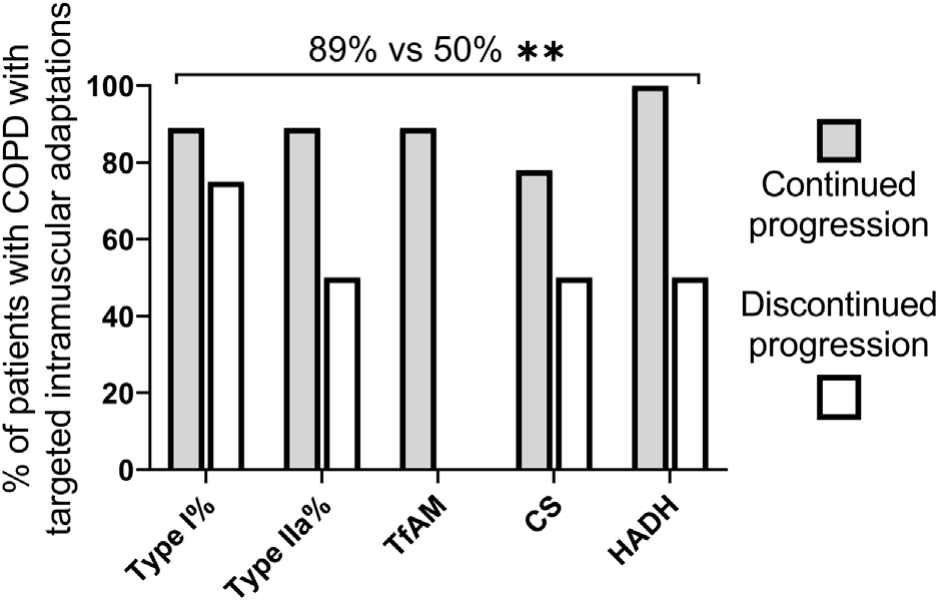
Median percentage of patients with COPD an increase in percentage of Type I fibres, TfAM, CS and HADH protein levels and a reduction in the proportion of Type IIa fibres as expected due to the design of the low-load resistance training regimen (American College of Sports Medicine, 2009). COPD = chronic obstructive pulmonary disease (COPD), CS = citrate synthase; HADH = hydroxyacyl-coenzyme A dehydrogenase; TfAM = mitochondrial transcription factor, W, Week* *p* < 0.05, ** *p* < 0.001.

At the individual level, a larger proportion of patients in the continued progression group (median 89%, range 78–100%) than in the discontinued progression group (median 50%, range 0–50%) demonstrated intramuscular adaptations consistent with the design of the low-load resistance training regimen, i.e., to improve the endurance capacity of the muscle. Additionally, after dichotomization, all nine patients in the continued progression group had a targeted change in at least four out of five intramuscular outcomes. In contrast, none in the discontinued progression group had a targeted change in more than three out of five intramuscular outcomes (*p* = 0.001) (Figure 5).

### 3.3 Muscle performance

**FIGURE 6:**
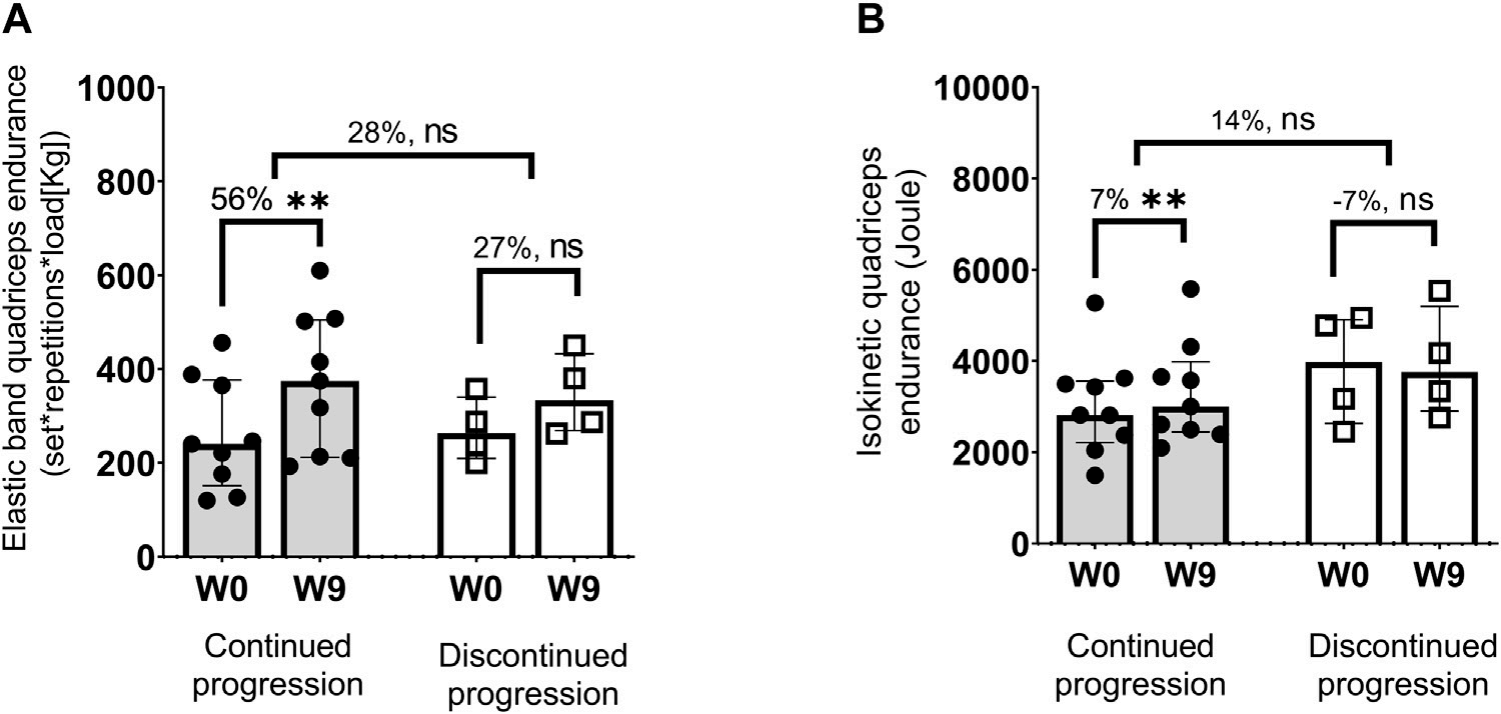
Values are Median (interquartile, 25–75 range). W = Week. Increase in elastic (set * repetition * load) **(A)** and isokinetic total work from 30 maximal isokinetic knee extensions performed at an angular velocity of 90°/second) **(B)**. * *p* < 0.05, ** *p* < 0.001.

The percentage changes in elastic band and isokinetic knee extension endurance was compared between groups. Only in the continued progression group did the increased elastic band and isokinetic quadriceps endurance reached statistical significance although there was no between-groups significant differences (median difference between groups range 14–29%, *p* = 0.330–1.000) (Figure 6).

### 3.4 Differences in training volume between the first and the last 4 week period and changes in muscle variables

For muscle variables that were significantly different between the continued and the discontinued progression groups, moderate to high associations were seen between the difference in training work during the first vs the last 4 weeks of training and changes in Type I fibers (r_s_ = 0.401), Type IIa fibers (r_s_ = −0.423), and TfAM protein levels (r_s_ = 0.527), although these correlation did not reach statistical significance (*p* = 0.067–0.176).

## 4 Discussion

The novel findings of the current study are that patients with COPD who had a continued progression of training volume across the 8-weeks intervention had an increased proportion of Type I fibers, and TfAM muscle protein levels and a decreased proportion of Type IIa fibers compared to those in whom training volume did not continue to progress after the initial weeks. Additionally, HADH muscle protein levels and quadriceps endurance only improved in the continued progression group, although no significant between-group differences were seen. Lastly, when analyzing individual responses, as recommended when the sample size is small (Dwivedi et al., 2017; Morgan, 2017), the vast majority of patients in the continued progression group (range 78–100% across selected outcomes) demonstrated intramuscular adaptations aligned with the objective of the low-load resistance training regimen (Kraemer and Ratamess, 2004; American College of Sports Medicine, 2009; Nyberg et al., 2015), which was not seen to the same extent in the discontinued progression group (range 0–50%). The consistency between the sub-group and individual responses give credibility to the findings despite the small sample size and post hoc nature of our analysis.

Resistance training is a cornerstone in COPD rehabilitation and the preferred strategy if the goal is to counteract the negative consequences of quadriceps dysfunction, increase muscle function and enable various morphological and structural adaptations (Iepsen et al., 2015b; Liao et al., 2015; De Brandt et al., 2016; Nyberg et al., 2016; De Brandt et al., 2018). The effect of resistance training on muscle function can be explained by a combination of both neural and intramuscular adaptations (Sale, 1988; Peterson et al., 2004; Peterson et al., 2011). It has also been suggested that neural factors account for the vast majority of gain in muscle function during the initial weeks of resistance training, while the relative importance of intramuscular adaptations increases over time (Sale, 1988; Folland and Williams, 2007; Škarabot et al., 2021). Furthermore, as previously highlighted, continued progression of training volume is one way to enable a gradual increase of muscle stress, which is of utmost importance for continuous improvements in muscle function and structure over time (Kraemer and Ratamess, 2004; American College of Sports Medicine, 2009).

Notably, there were no difference in total training volume between the continued and the discontinued progression group. Thus, taken together, our results support and extend previous observations (Sale, 1988; Folland and Williams, 2007; Škarabot et al., 2021) by suggesting that training volume, and specifically the continued progression of training volume after the initial weeks of training, is necessary to enable morphological and structural adaptations and thus, counteract quadriceps dysfunction within the COPD population. These findings are relevant to COPD management considering the high prevalence and adverse consequences of quadriceps dysfunction in this population (Swallow et al., 2007; Maltais et al., 2014; Frykholm, 2020; Géphine et al., 2021).

Direct comparison of our findings to previous work is difficult since, to our knowledge, this is the first study to investigate the impact of training volume and, specifically role of continued progression of training volume following low-intensity resistance exercises in patients with COPD. However, with aerobic training modalities, the importance of continued progression of training volume was nicely illustrated by Bjorgen et al. (2009), comparing one-leg to two-legged cycling in patients with COPD. These investigators reported that the two-legged trained group stopped progressing their training volume after the initial weeks of training. In contrast, the one-legged trained group was able to continue to increase their training volume across their 8-weeks intervention period and, as a result, also exhibited larger training effects. Similar findings were also seen in studies by Dolmage et al. (Dolmage and Goldstein, 2006; Dolmage and Goldstein, 2008; Evans et al., 2015). Furthermore, Casaburi et al. (1991) demonstrated that exercise work rates determined the size of the training effect in patients with COPD while Bronstad et al. (2012) demonstrated that continued progression of exercise volume over a 6-weeks knee extensor exercise intervention led to multiple intramuscular adaptations, including improved peak quadriceps muscle oxygen uptake and maximal mitochondrial respiration suggesting that optimizing the load on the quadriceps muscle over time as it is one of the key factors determining the training response (Casaburi et al., 1991; Kraemer and Ratamess, 2004; American College of Sports Medicine, 2009; Bronstad et al., 2012).

In this post hoc analysis, the study population was retrospectively divided into two groups according to the ability of increasing training volume. Although not statistically significant, the associations seen between the difference in work performed during the first 4 weeks (week 1–4) versus the last 4 weeks (week 5–8) of training and the change in Type I and Type IIa fibers and in TfAM muscle protein levels were consistent with the dichotomized analysis, thus providing reassurance about the validity of the findings. Lastly, it was observed that the number of responders was significantly different between the continued and the discontinued group (Figure 5). Since optimizing training volume is a key modifiable strategy to reduce training response heterogeneity and the number of low responders following progressive resistance training in older healthy adults (Lavin et al., 2019), we hypothesized that the low number of responders in the discontinued group is a result of the inability for these patients to tolerate progressive overload over time.

### 4.1 Methodological considerations

When interpreting our findings, several methodological considerations need to be made. First of all, even though similar or even smaller COPD sample sizes have been used in previous work on intramuscular adaptations following exercise training in COPD (De Brandt et al., 2016), the study’s primary limitation is the small sample size and the post hoc nature of our analysis potentially limiting the reliability and validity of our findings. As such, the results should be interpreted cautiously. To increase the validity and interpretation of our findings and line with recommendations for small sample size analysis (Dwivedi et al., 2017; Morgan, 2017), we decided to visualize group median values and individual data points for each analysis (Figures 3, 4, 6). According to this approach, our results indicate that the vast majority of patients in whom training volume continued to increase after the initial weeks of training demonstrated multiple intramuscular adaptations such as an increase in the percentage of Type I fibers, an increase in TfAM, CS and HADH muscle protein levels, and a reduction in the proportion of Type IIa fibers aligned with the objective of the resistance training approach (American College of Sports Medicine, 2009), increasing the validity of the findings despite the small sample size. Moreover, the small sample size may also influence the reliability of our findings, e.g., due to low statistical power (Button et al., 2013). Notably, statistically significant differences were observed on several of the included outcomes indicating sufficient statistical power. Additionally, the consistency between the sub-group and individual responses give credibility to the findings despite the small sample size and post hoc nature of our analysis. Still, low statistical power is likely a key factor for the associations between the difference in training work during the first vs the last 4 weeks of training and changes in Type I fibers (rs = 0.401), Type IIa fibers (rs = −0.423), and TfAM protein levels (rs = 0.527), being insignificant despite being moderate to high. Notably, the current study used a low-load resistance training design. It is not certain that results would have been similar if a high-load resistance training regimen had been used, even if research is healthy older adults suggests that it should be the case (Lavin et al., 2019).

Moreover, controlling for factors such as differences in personal characteristics that could confound our findings was not possible due to the small sample size and it is thus unclear why some patients were able to tolerate progressively greater training volume after the initial weeks of training while others reached a plateau. Notably, as highlighted in a recent narrative review, multiple mechanisms likely underlie the development of plateaus during exercise training, presenting a significant challenge in both healthy subjects and patients with chronic diseases (Gelman et al., 2022). In the current study, no apparent between-group differences were seen in potentially confounding factors such as patient characteristics, lung function measurements, exercise capacity, fiber type distribution or oxidative muscle protein levels at baseline, or adherence to the training interventions, that could explain our findings. Other factors, not measured within the current trial, may have contributed to the presence of a plateau in training volume in the discontinued progression group. For example, the present study did not include a direct assessment of muscle activation, motor unit conduction velocity, contractile twitch properties, changes in sensory receptors reducing inhibition, or reduced antagonist activity (Gabriel et al., 2006; Jenkins et al., 2017; Škarabot et al., 2021). Considering that neural adaptations tend to occur in the early weeks of training, reaching a plateau thereafter (Gabriel et al., 2006; Jenkins et al., 2017; Škarabot et al., 2021; Gelman et al., 2022), it could be hypothesized that patients in the discontinued progression group had early neural adaptations followed by plateau in neural adaptations. However, since neural adaptations were not measured in the current trial, this could not be confirmed. Notably, patients of the discontinued progression group might have benefited from introducing variations in the training program (Kraemer and Ratamess, 2004; American College of Sports Medicine, 2009). Optimization of training volume over time could be achieved by incorporating variation, or periodization in the number of sets, repetitions or load during the training program (Kraemer and Ratamess, 2004; American College of Sports Medicine, 2009). Using periodization is also a practical approach to resistance training that may be used to maximize gains (Signorile et al., 2005). Still, whether or not adding periodization would have enabled those in the discontinued group to continue to increase their training volume, and optimally overload the muscle remains to be determined. Thus, subsequent larger-scale investigations are needed to confirm or refute our findings, enabling control of potential confounding factors, preferably including other exercise modalities and measuring neural training adaptations (Gabriel et al., 2006; Jenkins et al., 2017; Škarabot et al., 2021).

### 4.2 Conclusion

In conclusion, continued progression of training volume after the initial weeks of training seems to be important to enable intramuscular adaptations following low-load resistance training among patients with severe to very severe COPD, and providing novel insight that could be helpful in the design of future resistance training studies to counteract quadriceps dysfunction within the COPD population. Still, considering the small sample size and the post-hoc nature of our analysis, these results should be interpreted cautiously, and further larger scale and prospective studies are warranted.

## Data Availability

The raw data supporting the conclusions of this article will be made available by the authors, without undue reservation.
